# Characterisation of GPR17‐expressing oligodendrocyte precursors in human ischaemic lesions and correlation with reactive glial responses

**DOI:** 10.1002/path.6381

**Published:** 2024-12-20

**Authors:** Stefano Raffaele, Bettina Hjelm Clausen, Francesca Carolina Mannella, Martin Wirenfeldt, Davide Marangon, Sarah Boe Tidgen, Silvia Corradini, Kirsten Madsen, Davide Lecca, Maria Pia Abbracchio, Kate Lykke Lambertsen, Marta Fumagalli

**Affiliations:** ^1^ Department of Pharmacological and Biomolecular Sciences ‘Rodolfo Paoletti’ Università degli Studi di Milano Milan Italy; ^2^ Department of Neurobiology Research, Institute of Molecular Medicine University of Southern Denmark Odense Denmark; ^3^ Department of Clinical Research, Brain Research – Inter Disciplinary Guided Excellence (BRIDGE) University of Southern Denmark Odense Denmark; ^4^ Odense Patient data Explorative Network (OPEN), Department of Clinical Research, Odense University Hospital University of Southern Denmark Odense Denmark; ^5^ Department of Pathology South Denmark University Hospital Odense Denmark; ^6^ Department of Pharmaceutical Sciences Università degli Studi di Milano Milan Italy; ^7^ Department of Cardiovascular and Renal Research, Institute of Molecular Medicine University of Southern Denmark Odense Denmark; ^8^ Department of Neurology Odense University Hospital Odense Denmark

**Keywords:** ischaemic stroke, GPR17 receptor, oligodendrocyte precursor cells, oligodendrocytes, microglia, astrocytes, remyelination, neuroinflammation, glial cell interactions, post‐mortem brain tissue

## Abstract

White matter damage and subsequent demyelination significantly contribute to long‐term functional impairment after ischaemic stroke. Identifying novel pharmacological targets to restore myelin integrity by promoting the maturation of oligodendrocyte precursor cells (OPCs) into new myelinating oligodendrocytes may open new perspectives for ischaemic stroke treatment. In this respect, previous studies highlighted the role of the G protein‐coupled membrane receptor 17 (GPR17) as a key regulator of OPC differentiation in experimental models of brain injury, including ischaemic stroke. To determine the translational value of GPR17 as a possible target in the context of human disease, we exploited immunohistochemistry to characterise the distribution of GPR17‐expressing cells in brain tissue samples from ischaemic stroke cases and correlated it with the reactive state of neighbouring glial cells. The results showed that GPR17 specifically decorates a subpopulation of differentiation‐committed OPCs, labelled by the peculiar marker breast carcinoma‐amplified sequence 1 (BCAS1), that accumulates in the peri‐infarct region in the later stages after the ischaemic event. Interestingly, the response of GPR17‐expressing cells appears to be paralleled by the switch of reactive microglia/macrophages from a phagocytic to a dystrophic phenotype and by astrocytic scar formation. A negative correlation was found between GPR17‐expressing OPCs and reactive microglia/macrophages and astrocytes surrounding chronic ischaemic lesions in female subjects, while the same relationship was less pronounced in males. These results were reinforced by bioinformatic analysis of a publicly available transcriptomic dataset, which implicated a possible role of inflammation and defective neuron‐to‐OPC communication in remyelination failure after ischaemic damage. Hence, these data strengthen the relevance of GPR17‐based remyelinating therapies for the treatment of ischaemic stroke. © 2024 The Author(s). *The Journal of Pathology* published by John Wiley & Sons Ltd on behalf of The Pathological Society of Great Britain and Ireland.

## Introduction

Ischaemic stroke is caused by the interruption of the blood supply to a specific area of the brain, representing the second most common cause of death in the European Union and a leading cause of permanent disability [1]. Current therapeutic options are limited to thrombolysis and thrombectomy, which have, however, a narrow treatment window and are associated with a high risk of cerebral haemorrhage [2]. On this basis, the development of novel strategies able to promote brain repair and functional recovery represents an urgent unmet medical need. An important consequence of ischaemic injury is the loss of oligodendrocytes (OLs); indeed, the resulting axonal demyelination is considered a major contributing factor to stroke‐associated neurological disability [3]. Therefore, fostering myelin reconstruction by supporting oligodendrocyte precursor cell (OPC) differentiation into new mature OLs has emerged as a promising approach to counter the progression of stroke‐related deficits [4].

Several studies have recognised the G protein‐coupled receptor 17 (GPR17) as a key regulator of OL differentiation [5]. GPR17 is highly expressed during the transition of proliferating OPCs to post‐mitotic cells expressing the OL marker O4 [5, 6], and it is then downregulated in fully mature cells expressing myelin basic protein, as demonstrated by different *in vitro* and *in vivo* studies [6, 7, 8, 9] and confirmed by single‐cell transcriptomics data [10, 11]. Noteworthy, a marked upregulation of GPR17 expression in OPCs close to demyelinated sites has been observed in several mouse models of central nervous system injury, including ischaemic stroke [5, 7, 12, 13, 14]. This is confirmed by fate‐mapping studies, demonstrating that the pool of GPR17‐expressing cells actively reacts to ischaemic brain damage by increasing their proliferation and migration towards the lesion and subsequently undergoes differentiation to restore myelin integrity [15, 16, 17]. Globally, these data indicate that, in rodents, GPR17 decorates a subset of OPCs committed to differentiation specifically involved in the reaction to myelin damage, setting the stage for the potential exploitation of this membrane receptor as a therapeutic target to promote remyelination after ischaemic stroke [18, 19]. In addition, it is now well established that the local inflammatory environment, dominated by reactive glial cells such as microglia and astrocytes, plays a major role in regulating remyelination after stroke [20, 21, 22, 23]. This is in line with our previous findings showing that reactive microglia are required for the recruitment of GPR17‐expressing OPCs towards the lesion at early stages after experimental stroke in mice, while at chronic time points microglia acquire detrimental and senescent‐like traits hampering the maturation of GPR17‐expressing cells [17, 21]. Therefore, understanding the functional interplay between microglia, astrocytes, and GPR17‐expressing OPCs at human ischaemic lesions may be critical to optimise the simultaneous targeting of GPR17 and the inflammatory milieu.

Currently, the clinical translation of GPR17‐targeted therapies is hampered by the lack of detailed studies investigating the spatiotemporal distribution of GPR17‐expressing cells in the brains of human ischaemic stroke cases and the possible correlation with disease progression and neighbouring glial cell reactivity. Here, we quantified GPR17‐expressing OPCs in human ischaemic brain lesions and correlated their distribution with allograft inflammatory factor 1 (AIF1)‐labelled microglia/macrophages and glial fibrillary acidic protein (GFAP)‐positive astrocytes in the same tissue, also evaluating the potential impact of disease stage, age, and sex on these parameters. We have provided the first evidence that GPR17‐expressing OPCs accumulate explicitly at the border of human ischaemic lesions and their density progressively increases over time after the ischaemic event, suggesting their involvement in long‐term remodelling of the damaged tissue. We then showed a dynamic spatiotemporal change in the reactivity of neighbouring microglia/macrophages and astrocytes after ischaemic injury, which correlates with the response of GPR17‐expressing OPCs surrounding chronic ischaemic lesions in a sex‐dependent manner. Lastly, bioinformatic analysis of a publicly available human transcriptomic dataset further validated the role of inflammation and defective neuron‐to‐OPC communication in remyelination failure in human ischaemic lesions. We envisage that these data will aid the development of GPR17‐targeted regenerative approaches for treating ischaemic stroke.

## Materials and methods

### Participants, recruitment, and data collection

This retrospective study comprised *n* = 34 autopsy specimens obtained from 27 patients with ischaemic stroke, divided into two cohorts.

Cohort 1 included *n* = 19 post‐mortem paraffin‐embedded brain tissue specimens from 14 patients with ischaemic stroke admitted to Odense University Hospital (OUH), Denmark in 2000–2005 and previously examined [24]. Within this cohort, there were nine males (median age 61 years) and five females (median age 78 years).

Cohort 2 consisted of *n* = 16 post‐mortem paraffin‐embedded brain tissue specimens from 13 patients archived at the Danish Brain Bank collection located at Brain Research – Inter Disciplinary Guided Excellence (BRIDGE), University of Southern Denmark, OUH, the Region Psychiatry of Southern Denmark, Odense, Denmark (https://www.sdu.dk/en/forskning/bridge/the-brain-collection, last accessed 14 October 2024). The selection was based on information obtained from histology and pathology reports stored on a GDPR‐secured server at the Open Patient data Explorative Network (OPEN), OUH and Department of Clinical Research, University of Southern Denmark, Odense, Denmark (https://open.rsyd.dk, last accessed 14 October 2024). A neuropathologist examined all brains to verify the diagnosis. In this cohort, there were eight males (median age 71 years) and five females (median age 69 years). Table 1 shows the sex, age at death, infarcted brain area, and infarct age.

**Table 1:** Clinical data from post‐mortem brain tissue.

Case no.	Sex	Age (years)	Infarcted brain area	Infarct age (days)	Cohort
1	M	76	Left temporal lobe	≥8	Cohort 1 (Clausen *et al* [24])
2	F	73	Right temporal lobe	≥8	
3	F	78	Right hemisphere	≤2	
4	M	67	Left temporal lobe	≥8	
5	M	61	Right parietal lobe	≥8	
6	F	83	Right hippocampus	3–7	
7	F	80	Right frontal lobe	≤2	
			Medulla	3–7	
			Pons	≤2	
			Striatum	≥8	
8	M	68	Left parietal lobe	≤2	
9	M	68	Caudate nucleus	≥8	
			Insula	≥8	
10	M	59	Right parietal lobe	≥8	
11	M	57	Left internal capsule	≥8	
12	F	67	Right occipital lobe	≤2	
13	M	38	Right parietal lobe	3–7	
14	M	48	Right temporal lobe	≥8	
15	M	82	Right corpus striatum	≥8	Cohort 2 (Danish Brain Bank collection)
16	F	69	Left corpus striatum	≥8	
17	M	74	Right corpus striatum	≥8	
18	M	80	Left frontal lobe, left hippocampus	≥8	
19	M	62	Mesencephalon	≥8	
20	F	74	Left frontal lobe	≥8	
			Left frontal gyrus	≥8	
21	M	82	Occipital lobe	≥8	
22	F	76	Right frontal lobe	3–7	
			Right frontal lobe	3–7	
23	M	60	Corpus striatum	≥8	
			Left corpus striatum	≥8	
24	F	68	Left corpus striatum	≥8	
25	M	64	Corpus striatum	≤2	
26	M	68	Left central lobe	≥8	
27	F	66	Left occipital lobe	≥8	

For all cases, data were collected on age at death, sex, the type and location of the ischaemic lesion, as well as the age of the lesion. The use of human post‐mortem brain tissue was approved by the Regional Committees on Health Research Ethics for Southern Denmark (Journal numbers S‐20220018, S‐20220072, and S‐20080042), and the project was reported to the Danish Data Protection Agency (https://www.datatilsynet.dk, GDPR number 16/34165). All data are hosted at OPEN (https://open.rsyd.dk/).

### Preparation of post‐mortem tissue

Human post‐mortem tissue encompassing infarcted brain tissue was formalin‐fixed, embedded in paraffin, and cut into 2‐ to 4‐μm‐thick serial sections on a microtome. Before staining, tissue sections were dewaxed in xylene and rehydrated in ethanol. For immunohistochemical staining, endogenous peroxidase activity was quenched using 1.5% v/v hydrogen peroxide in Tris‐buffered saline. For optimal staining protocols, heat‐induced epitope retrieval was performed using T‐EG buffer (10 mm Tris, 0.5 mm EGTA, pH 9) for chromogen staining, and citrate buffer (10 mm citrate, pH 6) for fluorescence staining.

### Haematoxylin and eosin and Luxol fast blue staining

For visualisation of nuclei and cytoplasmic inclusions, one section from each specimen was stained using haematoxylin and eosin (H&E) and for myelin visualisation, one section from each specimen was stained with Luxol fast blue (LFB) following standard protocols [24].

### Immunofluorescence

Before immunofluorescence staining, sections were bleached in Autofluorescence Eliminator Reagent (Merck Millipore, Merck KGaA, Darmstadt, Germany) following the manufacturer's guidelines. Sections were incubated overnight at 4 °C with the following primary antibodies: rabbit anti‐GPR17 (1:300; custom‐made by PRIMM, Milan, Italy) [25], mouse anti‐AIF1 (1:1,000; Sigma‐Aldrich, Merck KGaA, Søborg, Denmark; Cat. No. SAB2702364, clone GT10312), mouse anti‐GFAP‐Cy3 (1:500; Sigma‐Aldrich, Merck KGaA; Cat. No. C9205, clone G‐A‐5), mouse anti‐CNPase (1:100; Santa Cruz Biotechnology Inc., Dallas, TX, USA; Cat. No. sc‐166558, clone H‐2), mouse anti‐BCAS1 (1:500, Santa Cruz Biotechnology Inc., Cat. No. sc‐136342, clone 5), mouse anti‐NeuN (1:100, Merck Millipore, Cat. No. MAB377, clone A60), mouse anti‐A2B5 (1:100; Abcam Limited, Cambridge, UK; Cat. No. ab53521, clone 105), or mouse anti‐VGluT2 (1:1,000; Santa Cruz Biotechnology Inc., Cat. No. sc‐377425). The following day, sections were incubated with the following secondary antibodies: goat anti‐rabbit Alexa Fluor 594 (1:400, Thermo Fisher Scientific Inc., Waltham, MA, USA; Cat. No. A‐11012), goat anti‐rabbit Alexa Fluor 488 (1:400, Thermo Fisher Scientific Inc., Cat. No. A‐11008), or goat anti‐mouse Alexa Fluor 488 (1:200, Thermo Fisher Scientific Inc., Cat. No. A‐11001) for 2 h at room temperature. Nuclei were stained with 4',6‐diamidine‐2'‐phenylindole dihydrochloride (DAPI, 1:1,000; Thermo Fisher Scientific Inc., Cat. No. D1306) before mounting the slides. Images were acquired at 40× objective magnification using a Nikon ECLIPSE Ti2 confocal microscope (Nikon, Tokyo, Japan).

### Immunohistochemistry

For GPR17 immunohistochemical staining, sections were fixed for 20 min in methanol containing 0.1% v/v hydrogen peroxide at −20 °C and then incubated overnight at 4 °C with the primary antibody (rabbit anti‐GPR17, custom PRIMM) [25]. The following day, sections were incubated for 1 h at room temperature with the ImmPRESS HRP reagent kit (Vector Laboratories, Burlingame, CA, USA) for cohort 1 or Dako EnVision®+ Dual Link System‐HRP (Dako, Agilent Technologies Denmark ApS, Glostrup, Denmark) for cohort 2, followed by incubation with the 3,3'‐diaminobenzidine (DAB) solution. Sections were dehydrated in increasing concentrations of ethanol, followed by xylene, and mounted with DePeX mounting medium. Immunohistochemical staining of rabbit anti‐AIF1 (1:1,000; Wako, FUJIFILM Wako Pure Chemical Corporation, Osaka, Japan; Cat. No. 019‐19741), rabbit anti‐GFAP (1:2,000; Dako, Cat. No. Z0334), mouse anti‐CD68 (1:100, Abcam, Cat. No. ab783, clone PG‐M1), and mouse anti‐VGluT2 (1:1,000; Santa Cruz Biotechnology Inc., Cat. No. sc‐377425) was performed using the Dako Autostainer platform (Dako), as previously published [24]. Nuclei were counterstained with haematoxylin before mounting.

### Quantitative analyses

Images of the brain sections were acquired using a NanoZoomer slide scanner (Hamamatsu Photonics K.K., Shizouka, Japan) and then analysed by an investigator unaware of the experimental grouping, using NDP.view2 software (Hamamatsu Photonics K.K., https://www.hamamatsu.com/jp/en/product/life-science-and-medical-systems/digital-slide-scanner/U12388-01.html). For each brain specimen, images of H&E‐ and LFB‐stained parallel sections were used to identify the lesion area and define the infarct core (IC), the peri‐infarct (PI) area (within 1 mm from the border of the IC), and the normal‐appearing tissue (NAT). This map was then reported on scanned images of parallel sections labelled with GPR17, AIF1, GFAP, or VGluT2 and utilised to guide the subsequent analysis (see supplementary material, Figure S1). Cells positive for GPR17, AIF1, or GFAP were manually counted in each region using the ‘cell counter’ tool of NDP.view2. Only cells with a clearly visible nucleus stained by haematoxylin were counted. The total area analysed in each region was measured using the ‘freehand region’ tool of NDP.view2. Data are presented as cell density (number of positive cells/mm^2^), calculated by normalising cell number on the total area analysed. For VGluT2 quantification, the DAB signal was converted to binary images, and the percentage of area positive for VGluT2 over the total area analysed in the IC, PI area, and NAT was measured using Fiji/ImageJ (National Institutes of Health, Bethesda, MD, USA; https://fiji.sc) [26].

### Morphological analyses

Morphological analysis of AIF1‐positive cells was carried out using the ‘particles analysis’ tool of Fiji/ImageJ [26, 27]. The DAB signal was converted to binary images and analysed using a minimum particle size of 75 μm to exclude incomplete cells and debris. Cell size, cell circularity, and elongation index were quantified.

### Gene set enrichment analyses

A published transcriptomic dataset obtained through microarray analysis of post‐mortem brain tissue from six ischaemic stroke cases [28] was used for bioinformatic analysis. A total of 998 significantly differentially expressed genes [DEGs; false discovery rate (FDR) *q* value < 0.05, |fold‐change|>1.5] between ischaemic lesions (including IC and PI area) and contralateral NAT were subjected to functional enrichment analysis using the web‐based Enrichr toolkit [29] with default settings. The CellMarker [30] and Panglao [31] databases, retrieving cell‐specific markers based on single‐cell transcriptomic studies, were considered to identify the enriched cell types affected by ischaemic stroke. The Gene Ontology (GO) Cellular Component [32] and Jensen COMPARTMENTS [33] databases were considered to define the enriched subcellular components affected by ischaemic stroke. The GO Biological Process [32] and WikiPathways [34] databases were considered to investigate the enriched biological processes and molecular pathways affected by stroke, respectively. Enrichment scores were expressed as odds ratio (OR), computed by Enrichr using the following formula: OR = (1.0 *× a × d*)/Math.max(1.0 *× b × c*, 1), where *a* are the overlapping genes, *b* are the genes in the annotated set – overlapping genes, *c* are the genes in the input set – overlapping genes, and *d* are the 20,000 genes (or total genes in the background) – genes in the annotated set – genes in the input set + overlapping genes. The higher the OR, the stronger the association of DEGs with the related term (cell type, cellular component, biological process, molecular pathway).

### Network analyses

Protein–protein interaction (PPI) network analysis was performed using NetworkAnalyst [35]. In PPI networks, nodes are proteins, while edges represent known interactions between the linked proteins. In brief, PPI network analysis was executed in three steps: (i) obtaining the list of DEGs with log_2_ FC > |0.6|; (ii) submitting the list to the STRING‐based [36] PPI network tool (confidence score cut‐off = 900); and (iii) choosing a zero‐order network. NetworkAnalyst identified the most critical nodes, called hub genes, based on their degree and betweenness centrality. Degree centrality is the number of connections a node has to other nodes, whereas betweenness centrality corresponds to the number of shortest paths passing through the node.

### Statistical analyses

Statistical analyses and graphical representation of the data were performed using Prism 9 software (GraphPad Software Inc., San Diego, CA, USA). A non‐parametric Mann–Whitney test was performed to compare two groups without a normal distribution, whereas the Kruskal–Wallis test, followed by Dunn's *post hoc* analyses, was used to compare more than two groups. The Spearman's rank correlation coefficient test assessed correlation between data without a normal distribution; the correlation coefficients (*r*_s_) were interpreted as reported previously [37]: little to no relationship, 0.00–0.25; fair relationship, 0.25–0.50; moderate to good relationship, 0.50–0.75; and good to excellent relationship, >0.75. Data were considered statistically significant when *p* < 0.05.

## Results

### GPR17 specifically labels differentiation‐committed oligodendrocyte precursors in human ischaemic stroke lesions

**Figure 1:**
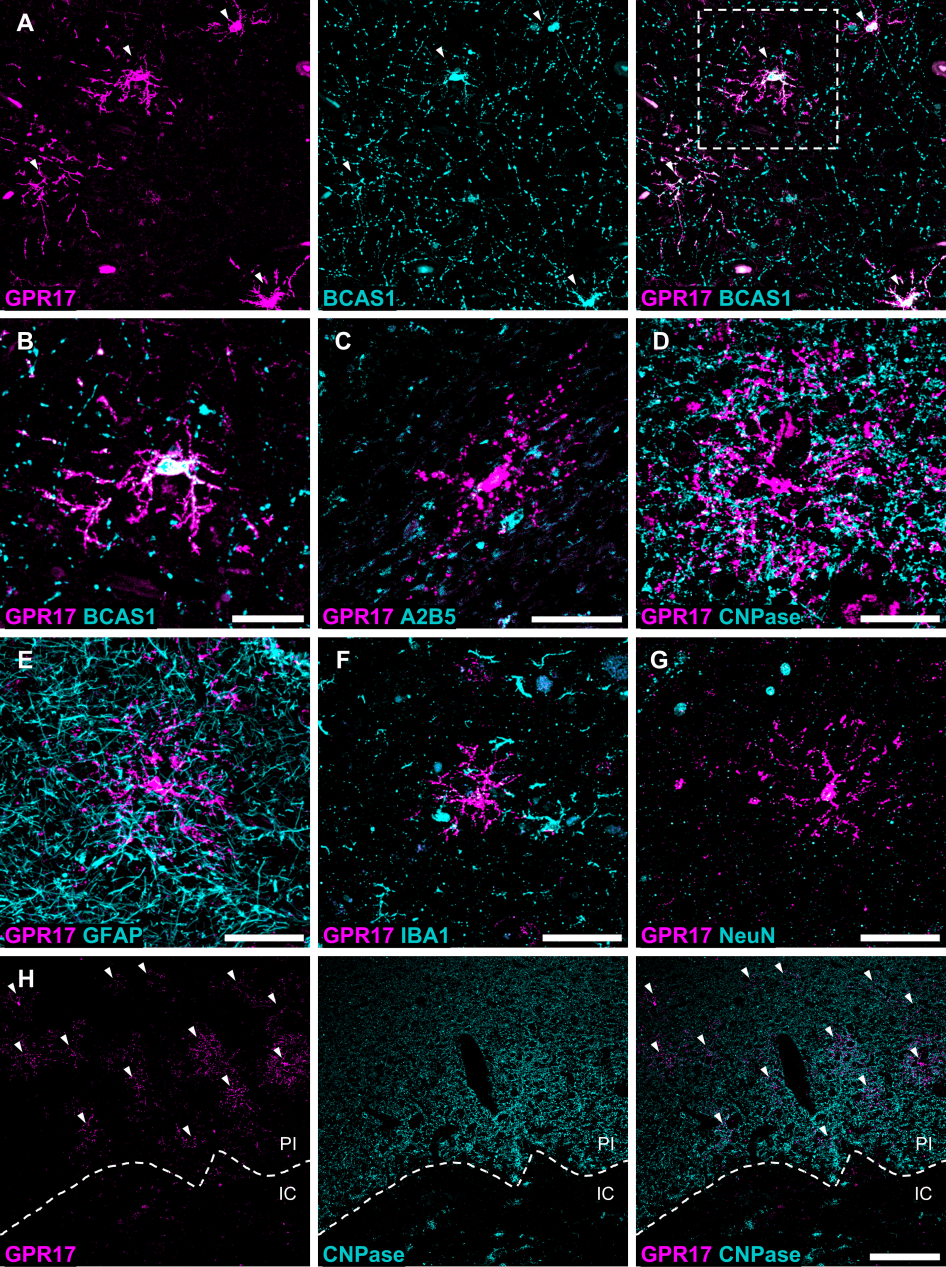
Characterisation of GPR17‐expressing cell identity in human ischaemic lesions. (A) Representative images of cells co‐expressing G protein‐coupled membrane receptor 17 (GPR17) (magenta) and the immature oligodendrocyte (OL) marker breast carcinoma amplified sequence 1 (BCAS1) (cyan), indicated by arrowheads, in post‐mortem human brain tissues from subjects affected by ischaemic stroke. The cell enclosed in the dashed white square is magnified in B. (B–D) Representative images of cells labelled for GPR17 (magenta) and the immature OL marker BCAS1 (B), the early OPC marker A2B5 (C), or the myelinating OL marker CNPase (D) in post‐mortem human brain tissues from subjects affected by ischaemic stroke (cyan). Scale bar: 50 μm. (E–G) Representative images of cells labelled for GPR17 (magenta) and the astrocyte marker GFAP (E), the microglia/macrophage marker AIF1 (also known as ionised calcium‐binding adapter molecule 1, IBA1) (F), or the neuronal marker NeuN (G) in post‐mortem human brain tissues from subjects affected by ischaemic stroke (cyan). Scale bar: 50 μm. (H) Representative images of cells labelled for GPR17 (magenta) and CNPase (cyan), showing accumulation of GPR17‐expressing cells (indicated by arrowheads) in the peri‐infarct (PI) area, while they are absent in the demyelinated ischaemic core (IC). PI area and IC are separated by the white dashed line. Scale bar: 200 μm.

To define the cellular identity and differentiation stage of GPR17‐expressing cells in human ischaemic stroke lesions, we performed double immunofluorescence labelling for GPR17 and markers of different OL maturation stages and other neuronal and glial cells. Notably, all the GPR17‐expressing cells detected were found to be also positive for BCAS1, which identifies the population of immature differentiation‐committed OPCs [11, 38] (Figure 1A,B). Conversely, no co‐localisation was highlighted between GPR17 and the early OPC marker A2B5 (Figure 1C), or between GPR17 and 2’,3’‐cyclic nucleotide 3’‐phosphodiesterase (CNPase), a marker of mature myelinating OLs (Figure 1D). These data suggest that, in human ischaemic tissue, GPR17 expression appears to be restricted to a specific intermediate stage of OL differentiation. In addition, no co‐localisation of GPR17 with the markers GFAP, AIF1, and NeuN was found, excluding the expression of the receptor in astrocytes, microglia/macrophages, and neurons, respectively (Figure 1E–G). Interestingly, these images allowed us to observe a marked accumulation of GPR17^+^ cells in the PI area (Figure 1H). On the contrary, GPR17^+^ cells were virtually absent in the IC, characterised by extensive demyelination indicated by loss of CNPase staining and by the presence of myelin debris positive for CNPase (Figure 1H).

### GPR17‐expressing cells accumulate in the peri‐infarct area at a late stage after ischaemic stroke

**Figure 2:**
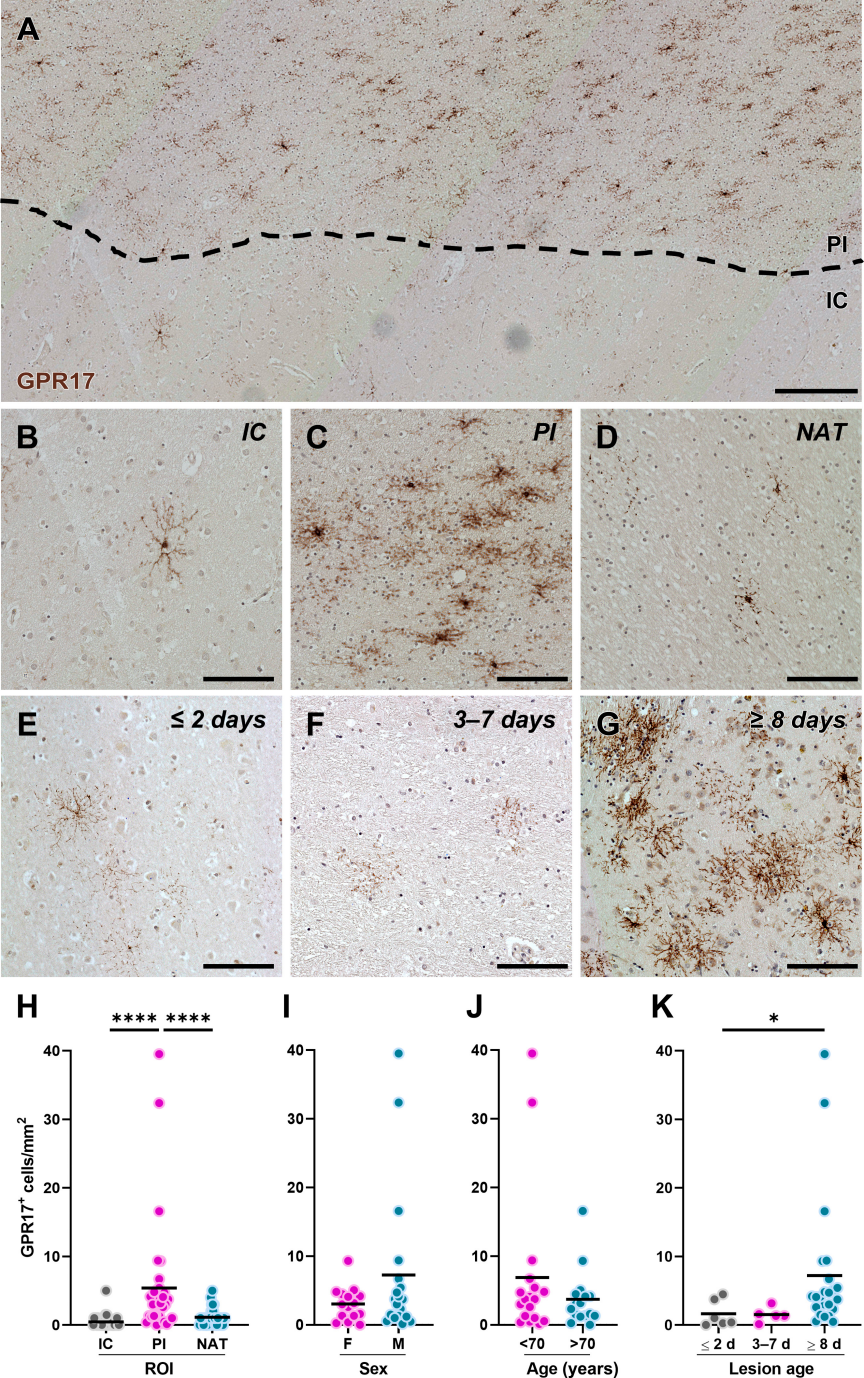
Distribution of GPR17‐expressing oligodendrocyte precursor cells (OPCs) in human ischaemic lesions. (A) Representative image of cells labelled for G protein‐coupled membrane receptor 17 (GPR17) in post‐mortem human brain tissue from a subject affected by ischaemic stroke. The black dashed line separates the infarct core (IC) and the peri‐infarct (PI) area. Scale bar: 250 μm. (B–D) Representative images of cells labelled for GPR17 in the IC (B), PI area (C), and normal‐appearing tissue (NAT) (D). Scale bar: 100 μm. (E–G) Representative images of cells labelled for GPR17 in the PI area of acute lesions (≤2 days; E), subacute lesions (3–7 days; F), and chronic lesions (≥8 days; G). Scale bar: 100 μm. (H) Quantification of GPR17‐expressing cell density in each region of interest (ROI), namely IC, PI area, and NAT, in post‐mortem human brain tissues from subjects affected by ischaemic stroke (*n* = 34). ****p < 0.0001, Kruskal–Wallis test followed by Dunn's *post hoc* analysis. (I and J) Quantification of GPR17‐expressing cell density in the PI region stratified by sex (I; females: *n* = 15; males: *n* = 19) and age (J; <70 years old: *n* = 18; >70 years old: *n* = 16) of the subjects. (K) Quantification of GPR17‐expressing cell density in the PI region stratified by the time elapsed since the ischaemic event (lesion age: ≤2 days, *n* = 6; 3–7 days, *n* = 5; ≥8 days, *n* = 23). *p < 0.05, Kruskal–Wallis test followed by Dunn's *post hoc* analysis.

The results described above suggest that the distribution of GPR17^+^ cells may depend on their localisation within the ischaemic tissue. We therefore performed immunohistochemistry to quantify the density of GPR17^+^ cells in different regions of interest (ROIs), namely the IC, the PI area, and the NAT distant from the lesion (see supplementary material, Figures S1 and S2). The results showed a significantly higher density of GPR17^+^ cells in the PI area compared with both the IC and the NAT (Figure 2A–D,H), suggesting that the regenerative response of immature OLs to ischaemic damage is localised at the border of the lesion. To evaluate possible differences in the density of GPR17^+^ cells in the PI area between specific subpopulations of cases, data were stratified based on the sex and age of the subjects. However, no significant differences were detected between female and male subjects (Figure 2I), or in individuals more than 70 years old compared with the younger age groups (Figure 2J). Finally, subjects were divided into three subpopulations based on the time elapsed since the onset of the ischaemic event: the first group included subjects with an acute infarct (≤2 days; Figure 2E); the second group included subjects in the subacute stage after stroke (3–7 days; Figure 2F); and the third group included subjects in the chronic stage after stroke (≥8 days; Figure 2G). This analysis unveiled a significant increase in the density of GPR17^+^ cells in the PI area of chronic lesions compared with acute ones (Figure 2E–G,K), suggesting that the GPR17^+^ immature OL population undergoes expansion over time after the ischaemic event.

### The reactivity of microglia/macrophages dynamically changes after ischaemic stroke

**Figure 3:**
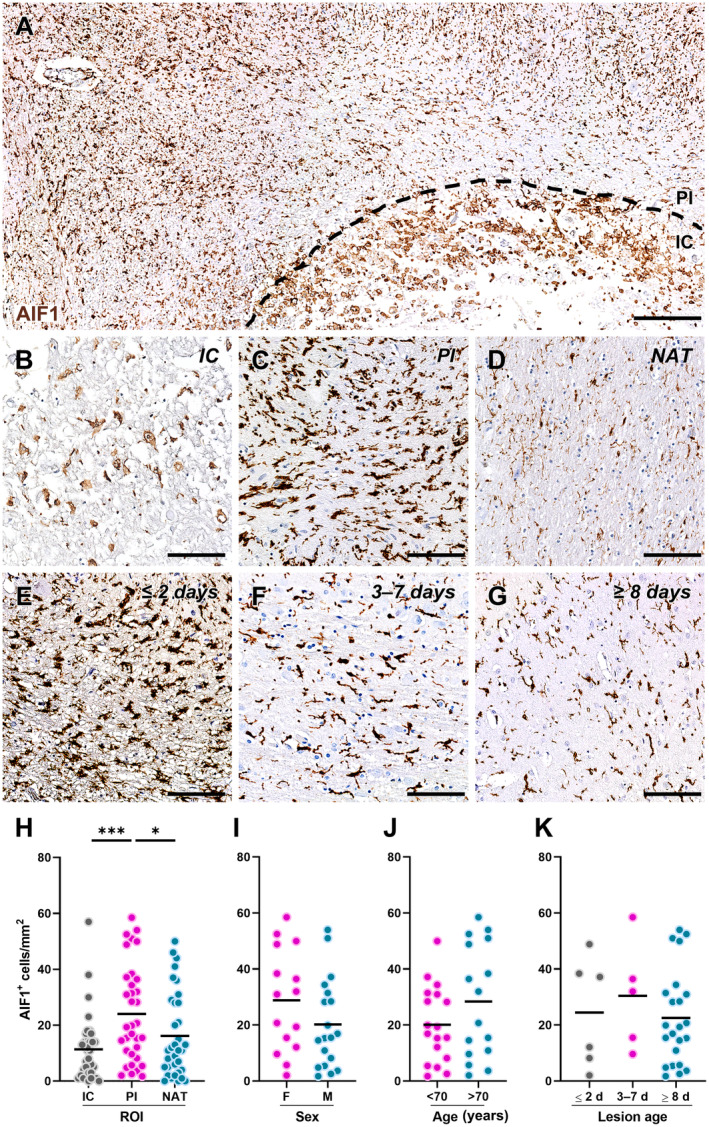
Distribution of allograft inflammatory factor 1 (AIF1)‐expressing microglia/macrophages in human ischaemic lesions. (A) Representative image of cells labelled for AIF1 in post‐mortem human brain tissue from a subject affected by ischaemic stroke. The black dashed line separates the infarct core (IC) and the peri‐infarct (PI) area. Scale bar: 250 μm. (B–D) Representative images of cells labelled for AIF1 in the IC (B), PI area (C), and normal‐appearing tissue (NAT) (D). Scale bar: 100 μm. (E–G) Representative images of cells labelled for AIF1 in the PI area of acute lesions (≤2 days; E), subacute lesions (3–7 days; F), and chronic lesions (≥8 days; G). Scale bar: 100 μm. (H) Quantification of AIF1‐expressing cell density in each region of interest (ROI), namely IC, PI area, and NAT, in post‐mortem human brain tissues from subjects affected by ischaemic stroke (*n* = 34). *p < 0.05, ***p < 0.001, Kruskal–Wallis test followed by Dunn's *post hoc* analysis. (I and J) Quantification of AIF1‐expressing cell density in the PI region stratified by sex (I; females: *n* = 15; males: *n* = 19) and age (J; <70 years old: *n* = 18; >70 years old: *n* = 16) of the subjects. (K) Quantification of AIF1‐expressing cell density in the PI region stratified by the time elapsed since the ischaemic event (lesion age: ≤2 days, *n* = 6; 3–7 days, *n* = 5; ≥8 days, *n* = 23).

Data from rodent models suggest that the response of GPR17‐expressing OPCs to ischaemic damage is tightly regulated by reactive microglia/macrophages [17, 21]. Thus, we quantified the density of AIF1^+^ microglia/macrophages in the IC, PI area, and NAT of human ischaemic tissue (Figure 3). The data showed a significantly higher density of AIF1^+^ cells in the PI area compared with the IC and NAT (Figure 3A–D,H). No differences were observed after stratification of the results based on the sex (Figure 3I), age (Figure 3J), and lesion age (Figure 3E–G,K) of the subjects.

**Figure 4:**
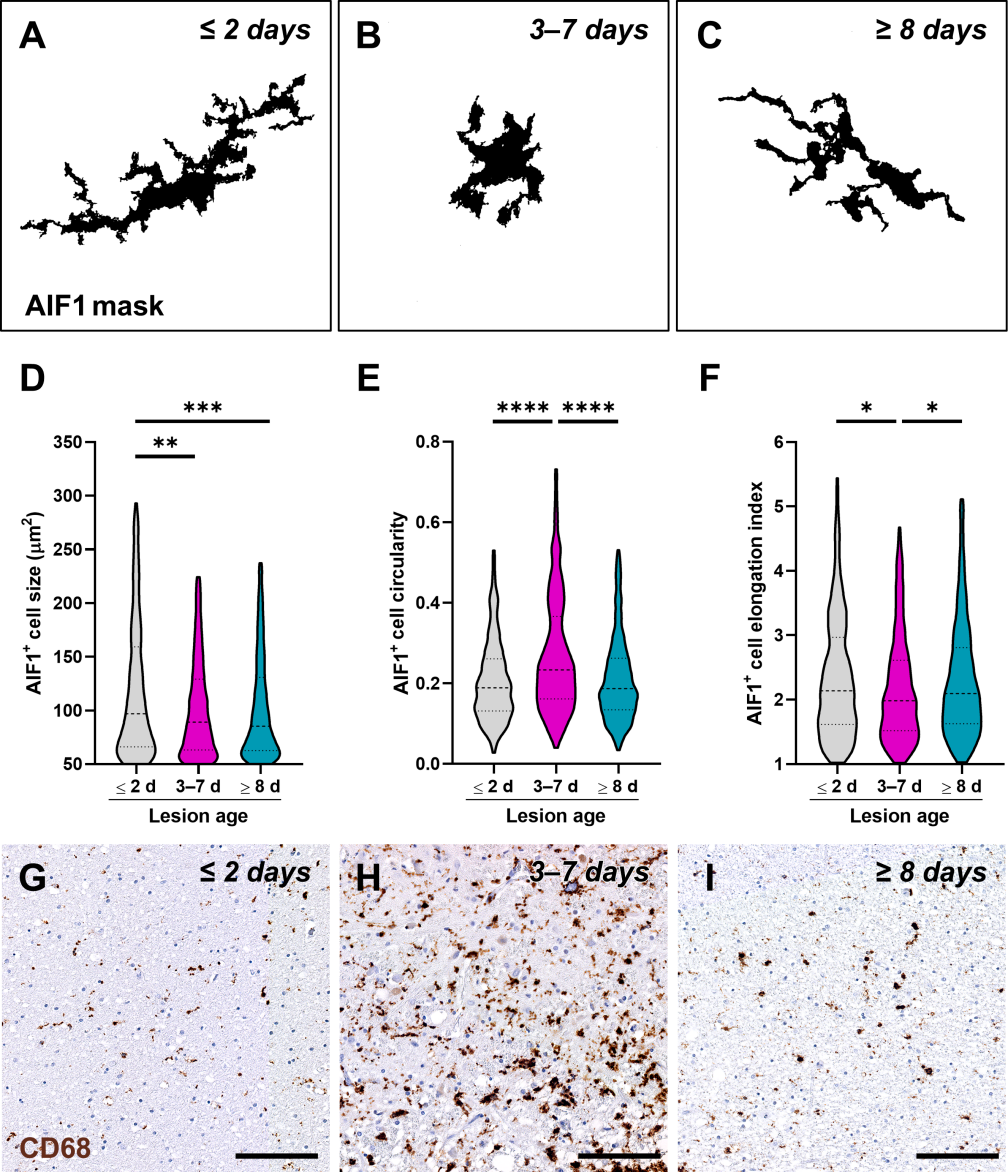
Morphological traits of allograft inflammatory factor 1 (AIF1)‐expressing microglia/macrophages in human ischaemic lesions. (A–C) Binary mask of cells labelled for AIF1 in the peri‐infarct (PI) area of post‐mortem human brain tissue collected ≤2 days (A), 3–7 days (B), and ≥8 days (C) after ischaemic stroke, used for morphological analysis. (D–F) Quantification of AIF1‐expressing cell size (D), circularity (E), and elongation index (F) in the PI region stratified by the time elapsed since the ischaemic event (lesion age: ≤2 days, *n* = 433; 3–7 days, *n* = 658; ≥8 days, *n* = 776). *p < 0.05, **p < 0.01, ***p < 0.001, ****p < 0.0001, Kruskal*–*Wallis test followed by Dunn's *post hoc* analysis. Dashed lines indicate median and quartiles. (G–I) Representative images of cells labelled for CD68 in the PI area of acute lesions (≤2 days; G), subacute lesions (3–7 days; H), and chronic lesions (≥8 days; I). Scale bar: 100 μm.

However, changes in the shape of reactive microglia/macrophages accumulating in the PI area at different stages after ischaemic stroke were observed (Figure 3E–G). To corroborate this observation, we performed morphometric analysis of AIF1^+^ cells in the PI area of acute, subacute, and chronic lesions (Figure 4A–C). This approach revealed a significantly larger AIF1^+^ cell size, as a proxy of cell reactivity, in acute lesions compared with subacute and chronic ones (Figure 4D). Conversely, AIF1^+^ cells surrounding subacute lesions were characterised by higher circularity, indicative of limited complexity of cell branching (Figure 4E), and by a lower elongation index, associated with reduced cell migratory capacity (Figure 4F), compared with their counterparts in acute and chronic lesions. Hence, these data unveiled dynamic changes in the response of microglia/macrophages in the PI area of human ischaemic lesions: at the acute stage, hypertrophic and elongated AIF1^+^ cells are recruited to the lesion (Figure 4A); in the subacute phase, AIF1^+^ cells switch to an amoeboid morphology resembling active phagocytes (Figure 4B); in the chronic phase, AIF1^+^ cells acquire a dystrophic morphology typical of senescent/dysfunctional cells [39], with reduced cell volume, fragmented cell processes, and ‘beaded’ spherical swellings (Figure 4C). These modifications are also reflected by the staining for the scavenger receptor CD68, indicative of the phagocytic activity of microglia/macrophages, which appears as tiny lysosomal dots in the acute stage (Figure 4G), is increased and expanded to the whole cell cytoplasm in the subacute phase (Figure 4H), and is mainly localised in small spherical protrusions in the chronic phase (Figure 4I).

### Marked reactive astrogliosis is evident in chronic ischaemic lesions

**Figure 5:**
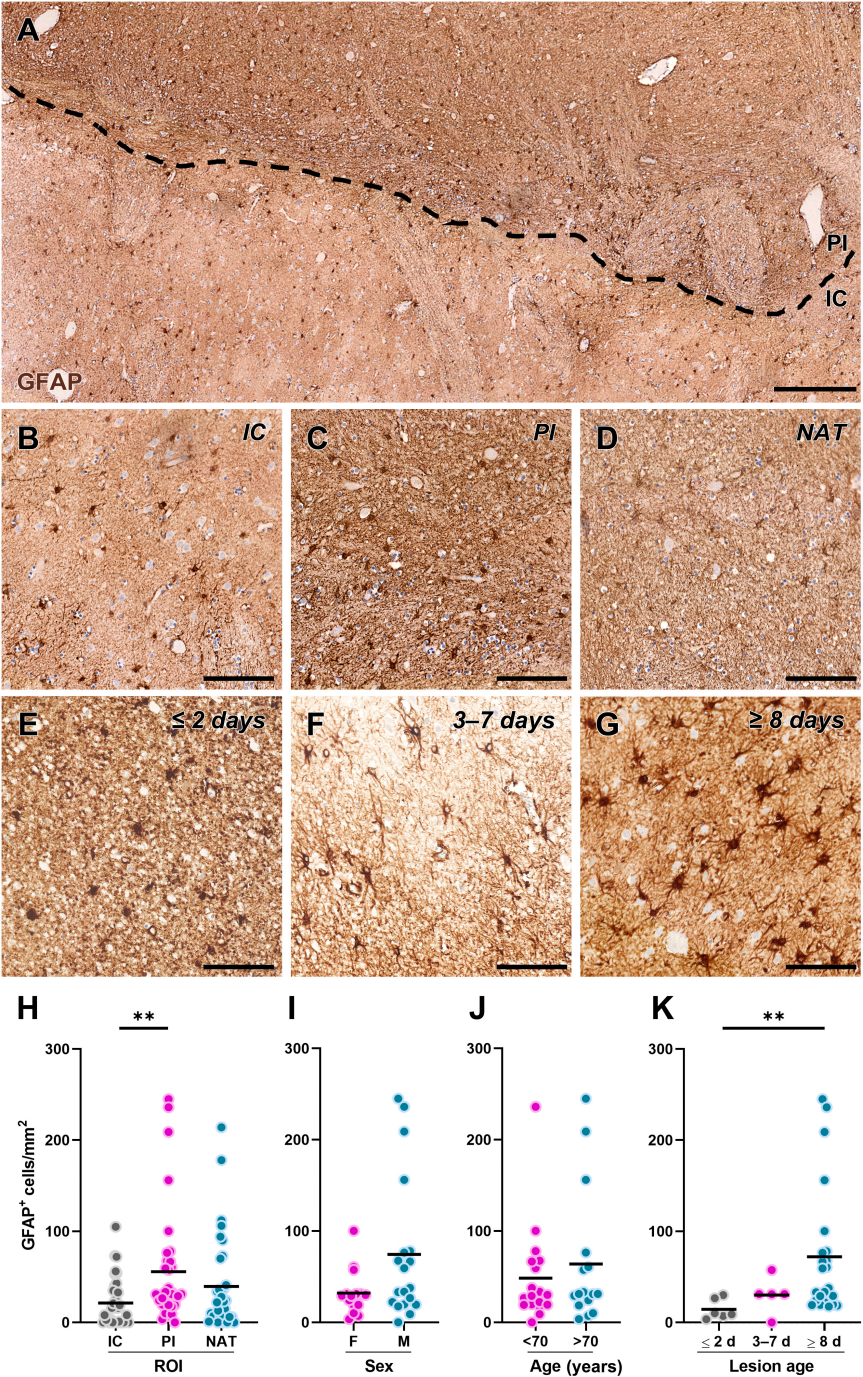
Distribution of glial fibrillary acidic protein (GFAP)‐expressing astrocytes in human ischaemic lesions. (A) Representative image of cells labelled for GFAP in post‐mortem human brain tissue from a subject affected by ischaemic stroke. The black dashed line separates the infarct core (IC) and the peri‐infarct (PI) area. Scale bar: 250 μm. (B–D) Representative images of cells labelled for GFAP in the IC (B), PI area (C), and normal‐appearing tissue (NAT; D). Scale bar: 100 μm. (E–G) Representative images of cells labelled for GFAP in the PI area of acute lesions (≤2 days; E), subacute lesions (3–7 days; F), and chronic lesions (≥8 days; G). Scale bar: 100 μm. (H) Quantification of GFAP‐expressing cell density in each region of interest (ROI), namely IC, PI area, and NAT, in post‐mortem human brain tissues from subjects affected by ischaemic stroke (*n* = 34). **p < 0.01, Kruskal–Wallis test followed by Dunn's *post hoc* analysis. (I and J) Quantification of GFAP‐expressing cell density in the PI region stratified by sex (I; females: *n* = 15; males: *n* = 19) and age (J; <70 years old: *n* = 18; >70 years old: *n* = 16) of the subjects. (K) Quantification of GFAP‐expressing cell density in the PI region stratified by the time elapsed since the ischaemic event (lesion age: ≤2 days, *n* = 6; 3–7 days, *n* = 5, ≥8 days, *n* = 23). **p < 0.01, Kruskal–Wallis test followed by Dunn's *post hoc* analysis.

Since reactive astrocytes have also been shown to affect remyelination after experimental stroke [23, 40], we next evaluated the spatiotemporal distribution of these glial cells in human ischaemic lesions using the GFAP marker (Figure 5). The results show a significantly higher density of GFAP^+^ cells in the PI area compared with the IC (Figure 5A–D,H). To identify any differences in the density of GFAP^+^ cells in the PI area in different subject populations, data were stratified based on the sex and age of the cases without, however, highlighting the significant effects of these parameters (Figure 5I,J). Finally, the distribution of GFAP^+^ cells in the PI area was evaluated based on the time elapsed since the onset of the ischaemic event (Figure 5E–G), revealing a significant increase in the density of GFAP^+^ cells in the PI area of chronic lesions compared with the acute ones (Figure 5K).

### The response of GPR17‐expressing OPCs surrounding chronic ischaemic lesions correlates with reactive glial cells in a sex‐dependent manner

The quantitative analyses described above indicate that the response of GPR17‐expressing OPCs and reactive glial cells is mainly localised in the PI area and dynamically changes over time after the ischaemic event. To obtain more insights into possible relationships between the different glial cells, we performed correlation analysis focusing on the PI area and lesions at the chronic stage after the ischaemic event when active tissue remodelling/regeneration is taking place based on our histological data (see supplementary material, Figure S2). Female and male subjects were analysed separately to highlight possible sexual dimorphism in these parameters. Regarding the GPR17^+^ and AIF1^+^ cell densities in female subjects, we observed a strong and statistically significant negative correlation, whereas no correlation was found in males (see supplementary material, Figure S2A). Similarly, we detected a strong negative correlation between GPR17^+^ and GFAP^+^ cell densities in females, while only a moderate negative correlation could be reported for males (see supplementary material, Figure S2B). Finally, a good positive correlation between AIF1^+^ and GFAP^+^ cells could be found in females, whereas no correlation was present in males (see supplementary material, Figure S2C). Hence, these data suggest that the response of GPR17‐expressing cells in chronic ischaemic lesions in female subjects might be hampered by the local reactivity of microglia/macrophages and astrocytes, while the same relationship may be absent in male cases.

Since the homeostatic and regenerative functions of reactive glial cells have been shown to decline with ageing [41], we then explored possible correlations between the age of the subjects and the densities of GPR17^+^, AIF1^+^, and GFAP^+^ cells in the PI area of chronic stroke lesions. In both males and females, there was no significant correlation between the age of the subjects and the density of GPR17^+^ cells (supplementary material, Figure S2D) and AIF1^+^ cells (supplementary material, Figure S2E), respectively. Interestingly, a positive correlation was found between the age and density of GFAP^+^ cells in male subjects, whereas no correlation was observed in females (supplementary material, Figure S2F), suggesting that the formation of a reactive glial scar around chronic lesions may be affected by the ageing process.

### The role of OPCs in tissue remodelling of human ischaemic lesions is confirmed by transcriptomics data

**Figure 6:**
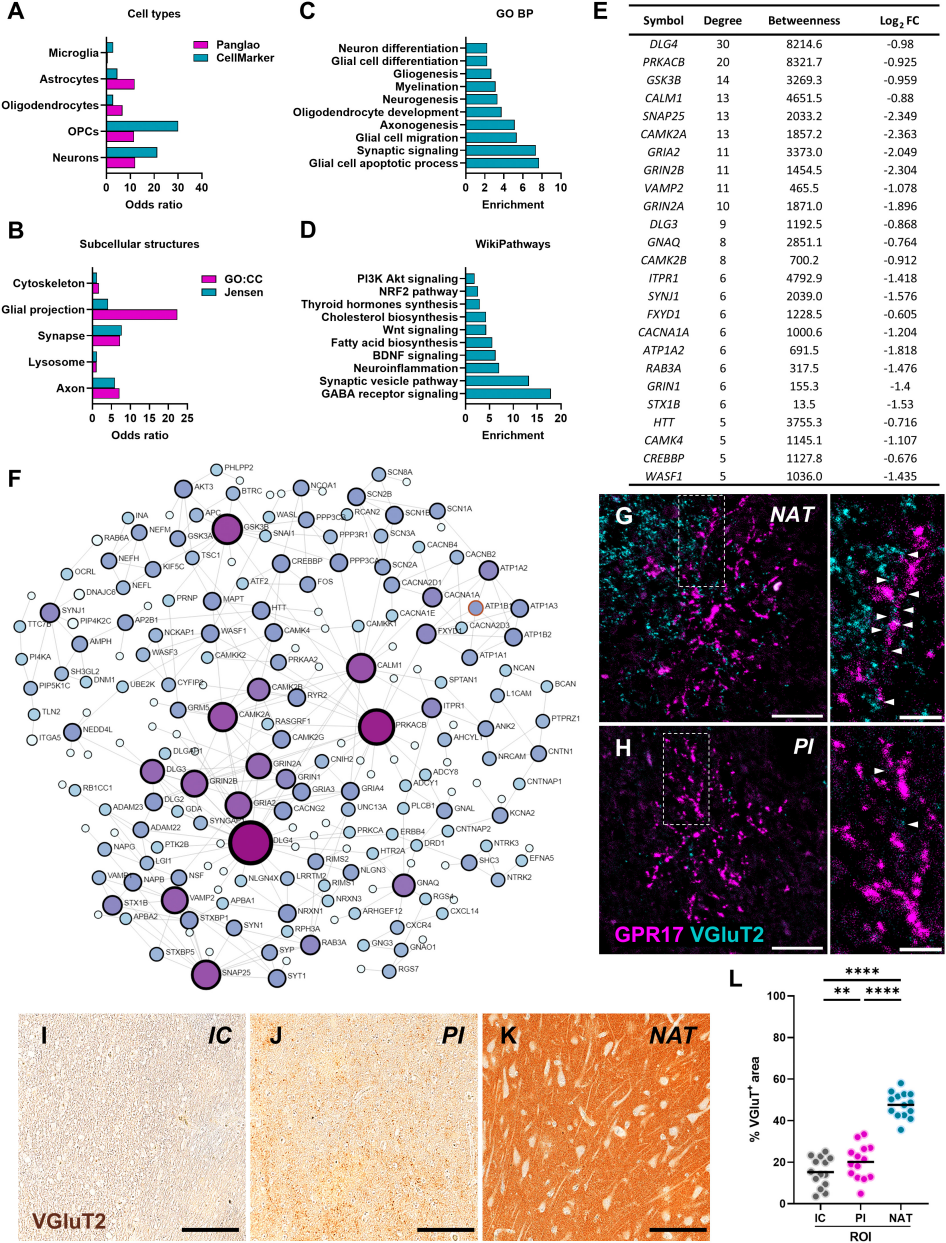
Enrichment and network analysis of differentially expressed genes between human ischaemic lesions and normal‐appearing tissues (NAT). (A–D) Cell types (A), subcellular structures (B), biological processes (C), and molecular pathways (D) significantly enriched in the dataset of differentially expressed genes (DEGs) between human ischaemic lesions and normal‐appearing tissue, emerging from the functional enrichment analysis. The over‐representation of each term in the DEG dataset is expressed as odds ratio. (E and F) Table showing the top hub genes of the network with their degree and betweenness centrality and log_2_ FC (E), and the zero‐order network (F) generated by means of the NetworkAnalyst software using DEGs with log_2_ FC > |0.6|. Protein–protein interactions have been constructed based on the STRING interactome (confidence score cut‐off = 900). The darker the colour, the greater the expression change. Node dimension directly correlates with the number of connections. An expanded view of panel F is available in supplementary material, Figure S3. GO, Gene Ontology; GO:CC, GO Cellular Component; GO:BP, GO Biological Process. (G and H) Representative images of cells labelled for GPR17 (magenta) and the synaptic protein VGluT2 (cyan) in the normal‐appearing tissue (NAT) and peri‐infarct (PI) area of post‐mortem human brain tissues from subjects affected by ischaemic stroke (scale bar: 30 μm). The cell branches in the dashed white boxes are magnified to highlight contacts between GPR17^+^ cells and VGluT2^+^ synapses, indicated by arrowheads (scale bar: 10 μm). (I–K) Representative images of VGluT2 labelling in the ischaemic core (IC) (I), PI area (J), and NAT (K). Scale bar: 100 μm. (L) Quantification of VGluT2‐positive area fraction in each region of interest (ROI), namely IC, PI area, and NAT, in post‐mortem human brain tissues from subjects affected by ischaemic stroke (*n* = 14). **p < 0.01, ****p < 0.0001, Kruskal–Wallis test followed by Dunn's *post hoc* analysis.

Despite the well‐characterised role of immature OPCs in brain repair and remyelination following ischaemic stroke in rodent models [3], it has been largely overlooked in human stroke pathology. To address this issue and corroborate the histological findings of our study, we took advantage of a previously published transcriptomics dataset comparing, in an unbiased manner, the bulk gene expression profiles of brain lesions (comprising both IC and PI areas) and contralateral NAT of six human subjects affected by ischaemic stroke [28]. In brief, the 998 significantly differentially expressed genes between the ischaemic lesion and NAT were subjected to functional enrichment analysis to investigate the cell types, cellular components, biological processes, and molecular pathways significantly dysregulated after stroke (Figure 6). The results reported below are mainly focused on glial cell responses to stroke, while a full report of the data emerging from this analysis is included in supplementary material, Table S1.

First, we considered two distinct databases retrieving cell‐specific markers altered in our dataset based on available single‐cell RNA‐sequencing data to estimate the cell types that are most affected by ischaemic stroke. Interestingly, OPCs and OLs emerged among the altered cell types after stroke, with a level of enrichment comparable to that of neurons, astrocytes, and microglia (Figure 6A). The analysis of the subcellular components using two independent databases revealed stroke‐related genes to be enriched not only in neuronal compartments, such as synapses, but also in axons and glial cell projections that are relevant for myelination (Figure 6B). The evaluation of the biological processes affected by ischaemic stroke revealed glial cell apoptosis and synaptic signalling among the most enriched terms but also highlighted several enriched terms related to OPC‐mediated remyelination, such as myelination, OL development, and glial cell migration and differentiation (Figure 6C). Accordingly, the analysis of the molecular pathways affected by stroke revealed several enriched terms related to synaptic signalling, inflammation, and myelination (Figure 6D), in line with the concept that a coordinated response of different glial cell types may be required to achieve effective remyelination of human ischaemic lesions.

Further bioinformatic analysis was performed to investigate PPI networks in our dataset and identify potential regulators of the biological effects induced by ischaemic stroke in humans (Figure 6E,F). According to the results of the enrichment analysis, the PPI network highlighted a large network of downregulated genes involved in postsynaptic signalling, including several glutamate receptor subunits as well as members of the membrane‐associated guanylate kinase family, regulating synaptic architecture and signal transduction (Figure 6E,F). Among these, it is worth highlighting the presence of genes involved in postsynaptic signalling, which may affect not only neuron–neuron but also neuron–glia communication. Interestingly, several of the downregulated hub postsynaptic components were found to be highly or exclusively expressed by OPCs compared with other cell types and have been implicated in neuronal activity‐dependent myelination processes [42, 43, 44, 45]. These findings led us to hypothesise that the loss of synaptic input at ischaemic lesions may contribute to the block of OPC maturation at the GPR17^+^ immature stage. In line with this hypothesis, we observed a dramatic loss of VGluT2^+^ synaptic contacts with GPR17^+^ cell branches in the PI area, where GPR17‐expressing OPCs accumulate, compared with the NAT (Figure 6G,H). This finding was also confirmed by the quantitative analysis showing a significant reduction of VGluT2^+^ area fraction in the IC and PI area compared with the NAT (Figure 6I–L). Hence, these data consolidate the involvement of OPCs in tissue remodelling after ischaemic stroke and suggest that synaptic dysfunction may contribute to remyelination failure of ischaemic lesions.

## Discussion

It is now well established that myelin loss represents a pivotal factor contributing to the neurological deficits associated with ischaemic stroke [4, 46]. After stroke, demyelination triggers a spontaneous reparative response mediated by OPCs in an attempt to form new myelin sheaths around denuded axons. In this context, previous evidence showed an increased abundance of GPR17‐expressing stalled OPCs in the proximity of the ischaemic lesion in murine models, raising interest in this receptor as an appealing druggable target for remyelinating therapy. Nevertheless, the lack of detailed characterisation of the type and distribution of GPR17‐expressing cells in human ischaemic brain lesions hampered the validation of this receptor as a pharmacological target in human pathology. Here, we fill this gap by uncovering that, in human ischaemic brain lesions, GPR17 labels explicitly a population of BCAS1‐expressing committed OPCs that have already exited their proliferating state but have not yet reached terminal maturation [10, 11], as confirmed by the lack of co‐localisation with the early OPC marker A2B5 and the mature marker CNPase. Importantly, these cells were found to accumulate in the PI area, a region of active tissue remodelling, compared with the necrotic IC and NAT. Similar results also emerged from a recent study, showing the greatest concentration of BCAS1^+^ cells accumulating in the PI area of human ischaemic brain tissue [47], and are in line with rodent studies which show OPC accumulation in the PI area after experimental brain ischaemia [15, 48]. This could mean that even in humans, following an ischaemic event, OPCs are recruited towards the damaged area. Still, most of them remain blocked in an immature GPR17^+^ differentiation stage, and only a small fraction manage to fully differentiate and generate myelin‐forming OLs.

These results, together with previous studies on human brain tissues in the context of other pathologies, such as traumatic brain injury and multiple sclerosis [25, 49], led to the consideration of GPR17 as a promising pharmacological target to promote remyelination in ischaemic stroke. In this respect, the pharmacological inhibition of GPR17 by montelukast (a drug already known for its anti‐asthmatic effect) was shown to reduce the extent of damage and restore brain connectivity and remyelination in ischaemic stroke mice [19]. On the other hand, RNAi and gene therapy approaches, directly reducing GPR17 expression at the mRNA level, also showed encouraging, but still preliminary, evidence [50, 51, 52].

Prolonged exposure to inflammatory cues, a hallmark of ischaemic injury, can hinder the downregulation of GPR17 in OPCs, thus impeding remyelination [53]. Conversely, pro‐regenerative microglial functions, including removing myelin debris, remodelling the extracellular matrix, and releasing soluble factors, support OPC recruitment and differentiation, implicating microglia as facilitators of remyelination [20, 21]. Moreover, astrocytes contribute to tissue remodelling and remyelination post‐stroke by forming the glial scar and releasing trophic factors [24, 54, 55]. Understanding the interplay between OPCs and reactive glial cells contributing to the inflammatory microenvironment of ischaemic lesions is thus critical for developing effective remyelinating strategies. Our results revealed a higher density of AIF1^+^ microglia/macrophages and GFAP^+^ astrocytes in the PI area, mirroring the distribution of GPR17^+^ cells. This observation, in agreement with previous studies [24, 56], suggests that glial reactivity is concentrated in regions of the ischaemic tissue undergoing active remodelling and may influence OPC recruitment and maturation. Interestingly, our results highlighted some important differences in the response of glial cells during the different phases following ischaemic damage. Specifically, a significantly higher density of GPR17^+^ and GFAP^+^ cells was observed in chronic lesions (≥8 days) compared with acute and subacute lesions. Conversely, for AIF1^+^ cell density, no significant differences were observed based on the infarct stage; however, morphological analysis showed dynamic changes in AIF1^+^ cell reactive phenotype in the PI area. During the acute phase post‐stroke, AIF1^+^ cells exhibited a hypertrophic and elongated morphology, indicative of increased cell reactivity. Subsequently, in the subacute stage, these cells switched to an amoeboid morphology typical of active phagocytes. Interestingly, the peak of phagocytic microglia/macrophages at the subacute disease stage in the PI area precedes the accumulation of GPR17^+^ cells, which is more evident at the chronic stage, when AIF1^+^ cells assumed a senescent‐like phenotype characterised by a dystrophic morphology [39]. Although it was not possible to perform a more detailed analysis of cell branching in three dimensions due to the type of samples available, the data suggested that microglia/macrophages may modify their reactive phenotypes during the evolution of human ischaemic lesions. Consistent with these findings, immunohistochemistry analysis showed increased CD68 expression during the subacute phase, compared with the other phases where CD68 expression is lower and localised in specific cell compartments. These results align with our previous work showing that early after experimental stroke in mice, microglia acquire a beneficial phenotype characterised by enhanced phagocytic capacity and reactive traits, which is necessary for the recruitment of GPR17‐expressing OPCs towards the ischaemic lesion [17]. On the contrary, at later stages after stroke, microglia lose their pro‐remyelinating functions in favour of an inflammatory and dystrophic state that hinders GPR17‐expressing OPC maturation [17]. Furthermore, our data agree with the classification of ischaemic lesions proposed by Zrzavy *et al* [56] and allow us, as a whole, to postulate a temporal sequence of events during the repair process of ischaemic lesions: (i) apoptosis of OLs induces demyelination; (ii) microglia acquire a reactive phenotype and begin to phagocytose myelin debris; (iii) finally, astrocytes begin to produce the glial scar, while the GPR17‐expressing OPCs attempt to undertake the remyelination process.

Considering recent studies pointing towards sexual dimorphism in glial cell responses after stroke [26, 57], we further investigated the possible relationships between GPR17^+^, AIF1^+^, and GFAP^+^ cells within the PI area of chronic lesions by performing sex‐specific correlation analyses on our cases. In females, we observed a negative correlation between GPR17^+^ cells and both AIF1^+^ and GFAP^+^ cells, suggesting a lower capability of beneficial glial reactive phenotypes in recruiting GPR17‐expressing OPCs and implying that a senescent‐like profile of microglia/macrophages and the formation of an astrocytic scar at the chronic stage may hinder the regenerative response of GPR17^+^ OPCs. Conversely, these correlations appear to be less prominent in males, underscoring the possibility of sex‐specific differences in the regulatory mechanisms governing interactions among OPCs, microglia/macrophages, and astrocytes after ischaemic stroke. These data also correlate with clinical results showing that women have poorer outcomes after stroke with higher rates of neurological deficits and a lower quality of life compared with men [58]. Such sexual dimorphism in the glial response to ischaemic stroke could be related to the type and availability of different hormones (i.e. oestrogen, progesterone, and testosterone), but sex chromosomal effects and epigenetic mechanisms have also been shown to play a role [58]. Further investigation, including a larger sample size, is needed to clarify these mechanisms and their implications for therapeutic interventions.

In addition to neuroinflammation, recent studies also suggest neuronal activity as a key driver of OPC maturation and myelin repair in a process that implicates the formation of glutamatergic synaptic connections between neurons and OPCs [43, 59, 60]. In this respect, our bioinformatic analysis and subsequent histological validation revealed a marked downregulation of OPC‐related postsynaptic components in human ischaemic lesions compared with normal tissue, which may be related to the loss of synaptic terminals due to either neuronal injury or a compensatory mechanism triggered by the increasing excitotoxicity. As a result, OPCs may lose the neuronal activity‐dependent pro‐differentiation ability, thus impairing their reparative response. Considering also recent data showing that neuron loss correlates with myelin deficits in human stroke lesions [4], these results corroborate a possible complex scenario in which a vicious circle of neuronal loss and myelination defects drives stroke‐related neurological disability. On this basis, a combination of neuroprotective and remyelinating approaches, together with immunomodulatory strategies improving microenvironment permissiveness, should be considered to foster functional recovery in stroke patients.

## Conclusions

Taken together, the data collected so far confirm that all three of the above‐mentioned glial cell populations (OLs, microglia, and astrocytes) are important for proper remyelination and efficient restoration of brain function after stroke. However, further studies are needed to better understand the role that glial cells play in human ischaemic stroke and the cellular mechanisms involved. Such studies should ideally increase the sample size and, where possible, reduce variability by selecting patients based on specific criteria considering comorbidities and lifestyle. Furthermore, advanced broad‐spectrum technologies, such as spatial or single‐cell transcriptomics, could prove fundamental to identifying new promising molecular targets and developing innovative therapeutic strategies for ischaemic stroke.

## Author contributions statement

SR, MW, KLL and MF conceived and designed the study. KLL and BHC performed histological and immunofluorescence experiments. SR, FCM and SC performed histological data analyses. SR and DM performed bioinformatic analyses. SBT identified ischaemic stroke cases from the Danish Brain Bank collection and assisted with histological experiments. KM assisted with tissue collection. SR and FCM wrote the manuscript, with input from KLL and MF. All experimental design, data analyses and manuscript preparation were overseen by DL, MPA, KLL and MF. All authors read and approved the final manuscript.

## Supporting information


**Figure S1.** Definition of the regions of interest (ROIs) for quantitative immunohistochemical analysis of human ischaemic lesions
**Figure S2**. Correlations between reactive glial cell populations in human ischaemic lesions and impact of ageing
**Figure S3**. Zero‐order network generated using NetworkAnalyst software for DEGs with log_2_ FC > |0.6|

**Table S1.** Expression analyses

## Data Availability

All human data are hosted at OPEN (https://open.rsyd.dk/), and requests to access datasets should be directed to martin.wirenfeldt.nielsen@rsyd.dk. The datasets used and/or analysed during the current study are available from the corresponding authors upon reasonable request.
