# Supplementary material for: Characterisation of GPR17‐expressing oligodendrocyte precursors in human ischaemic lesions and correlation with reactive glial responses
Source: J Pathol. 2024 Dec 20;265(2):226–43. doi: 10.1002/path.6381 (PMC11717493; doi:10.1002/path.6381)
Supplement: Supplementary file 1 — Figure S1. Definition of the regions of interest (ROIs) for quantitative immunohistochemical analysis of human ischaemic lesions Figure S2. Correlations between reactive glial cell populations in human ischaemic lesions and impact of ageing Figure S3. Zero‐order network generated using NetworkAnalyst software for DEGs with log2 FC > |0.6| [file PATH-265-226-s001.docx]

**Characterisation of GPR17-expressing oligodendrocyte precursors in human ischaemic lesions and correlation with reactive glial responses**

S Raffaele *et al. J Pathol.* <https://doi.org/10.1002/path.6381>

**Supplementary Figures S1–S3**


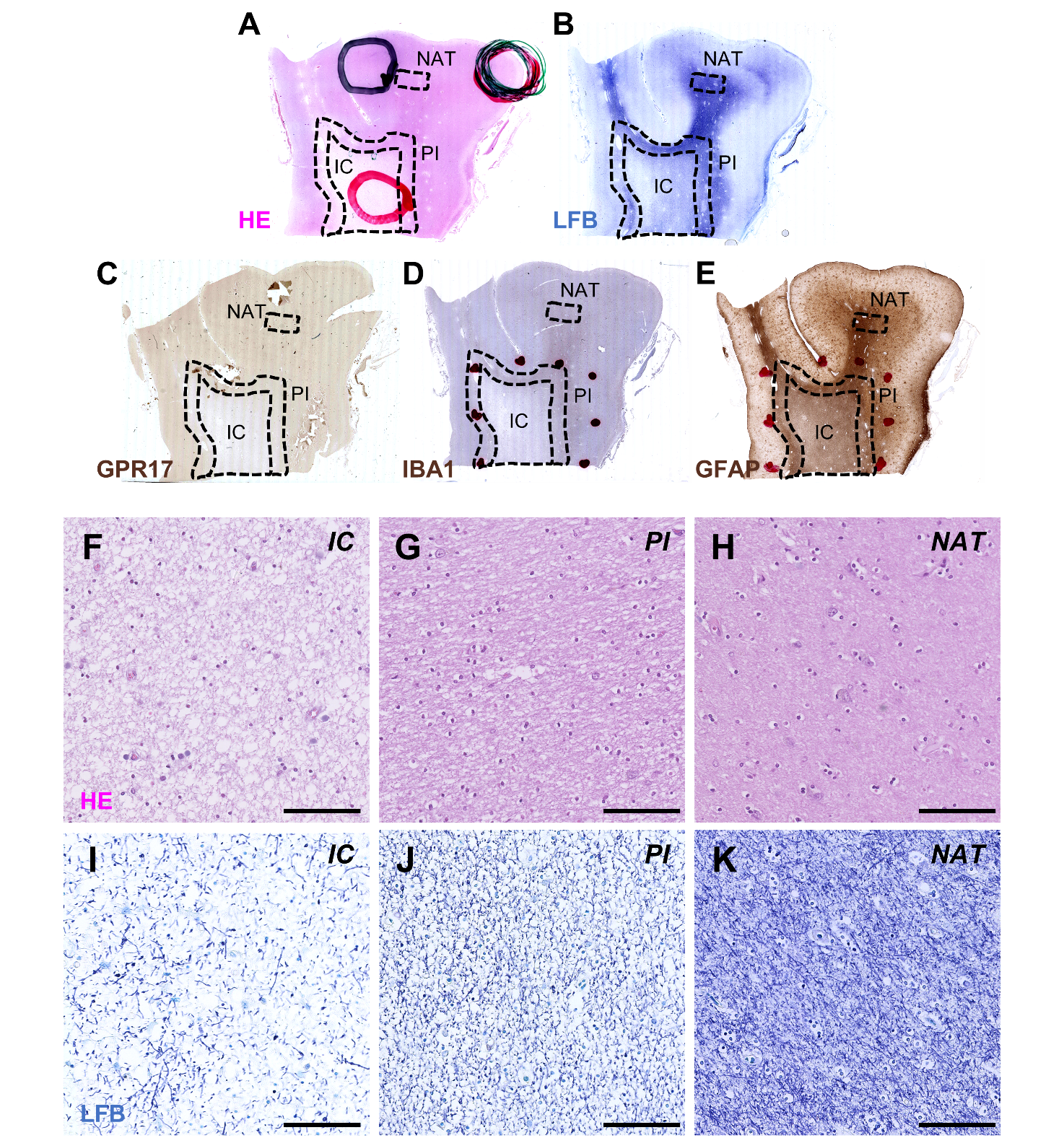


**Figure S1. Definition of the regions of interest (ROIs) for quantitative immunohistochemical analysis of human ischaemic lesions**. (A–E) Representative images of parallel sections of post-mortem human brain tissues from a subject affected by cerebral ischaemia stained for haematoxylin and eosin (HE; A), Luxol fast blue (LFB; B), GPR17 (C), AIF1 (also known as IBA1) (D), and GFAP (E). Black dashed lines define the infarct core (IC), peri-infarct (PI) area, and normal-appearing tissue (NAT) distant from the lesion. (F–H) Representative images of HE labelling in the IC (F), PI area (G), and NAT (H). Scale bar: 100 µm. (I–K) Representative images of LFB labelling in the IC (I), PI area (J), and NAT (K). Scale bar: 100 µm.


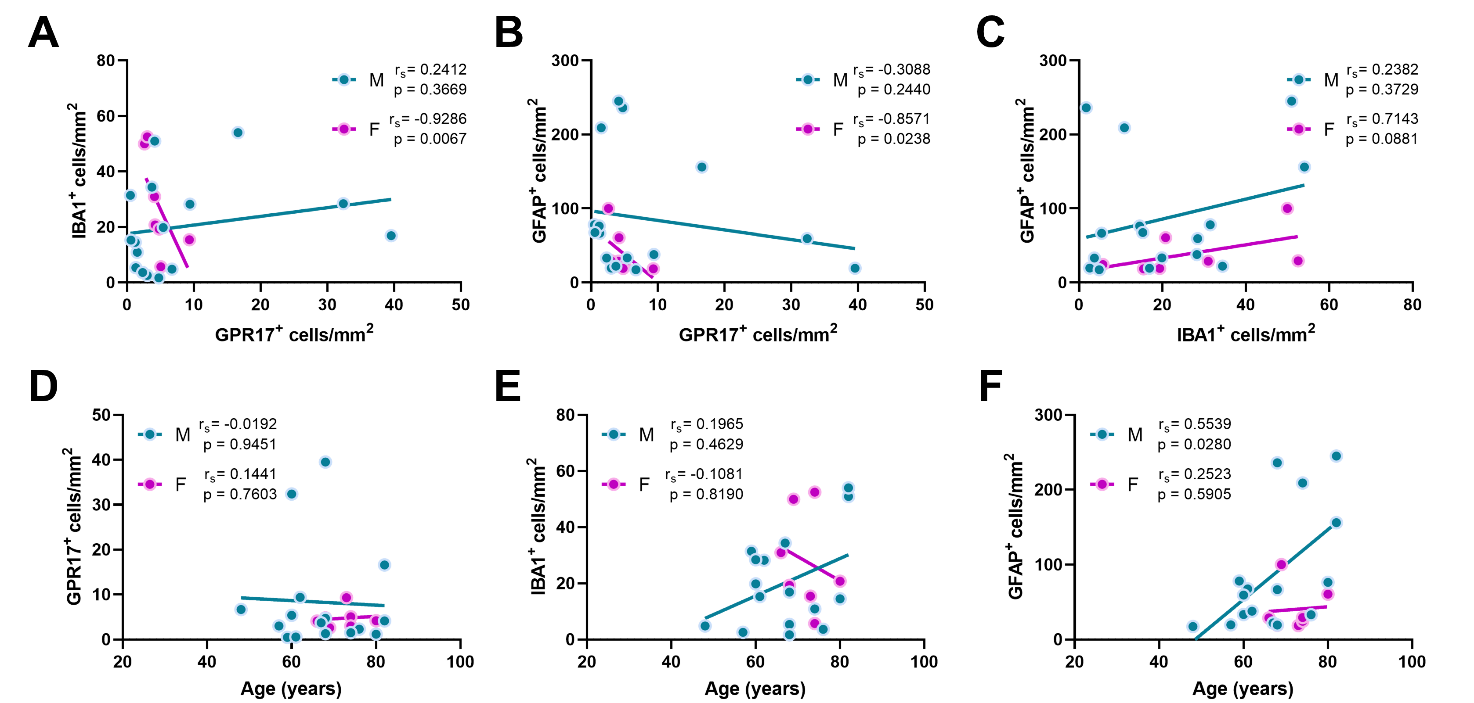


**Figure S2. Correlations between reactive glial cell populations in human ischaemic lesions and impact of ageing.** (A–C) Scatterplot representation of the linear correlation between the densities of GPR17^+^ and AIF1^+^ cells (A), GPR17^+^ and GFAP^+^ cells (B), and AIF1^+^ and GFAP^+^ cells (C) in the peri-infarct (PI) area of male (M) and female (F) human ischaemic stroke cases at the chronic stage (≥8 days; M: *n*= 16; F: *n*= 7). (AIF1 is also known as IBA1). For correlation analysis, a two-tailed Spearman’s rank correlation coefficient test was used (the correlation coefficient *r*_s_ and *p* value are reported in each graph). (D–F) Scatterplot representation of the linear correlation between the age of the subjects and the density of GPR17^+^ cells (D), AIF1^+^ cells (E), and GFAP^+^ cells (F) in the PI area of male (M) and female (F) human ischaemic stroke cases at the chronic stage (≥8 days; M: *n*= 16; F: *n*= 7). For correlation analysis, a two-tailed Spearman’s rank correlation coefficient test was used (the correlation coefficient *r*_s_ and *p* value are reported in each graph).


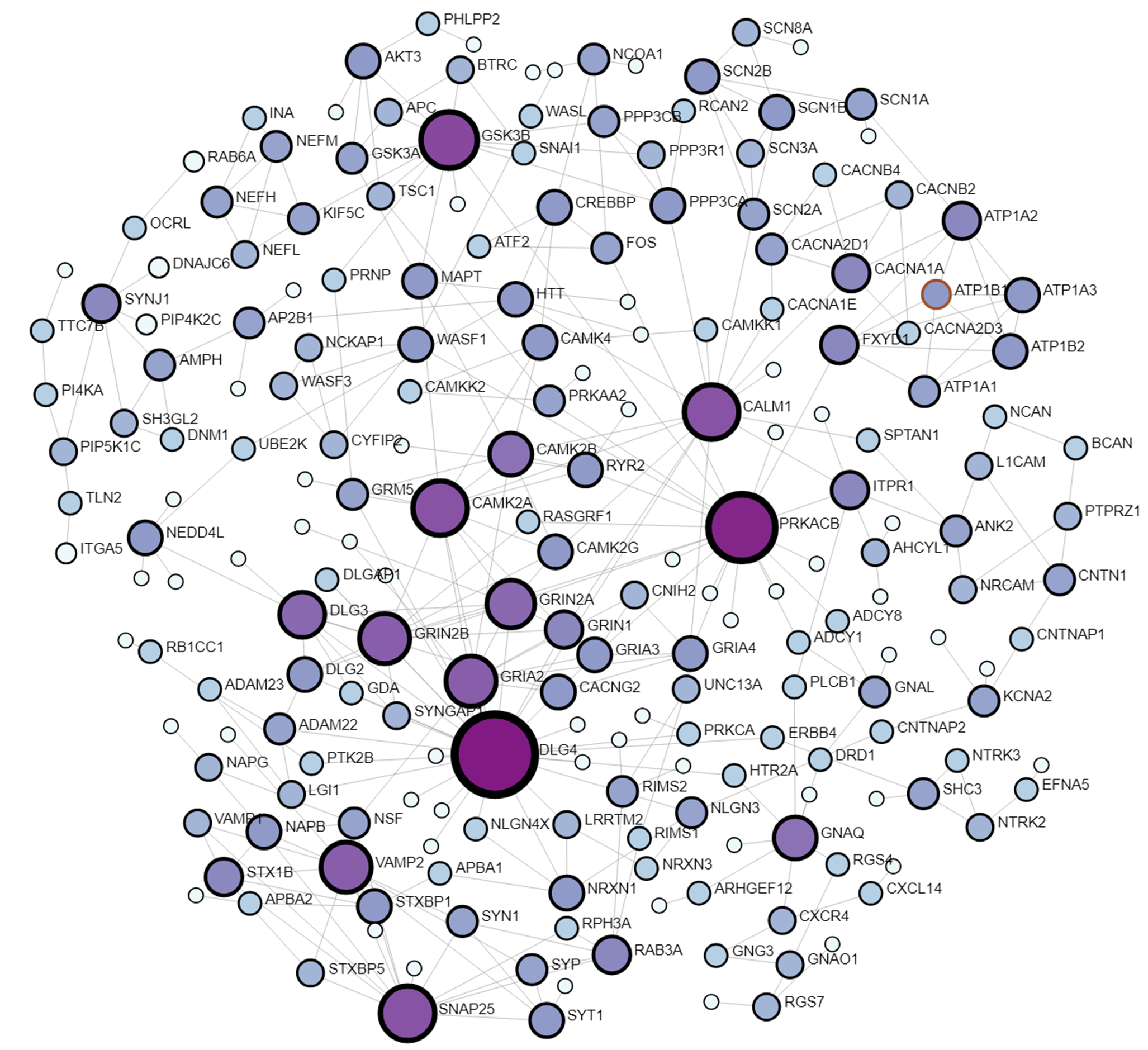


**Figure S3. Zero-order network generated using NetworkAnalyst software for DEGs with log_2_** **FC > |0.6|.** Protein–protein interactions have been constructed based on the STRING interactome (confidence score cut-off = 900). The darker the colour, the greater the expression change. Node dimension directly correlates with the number of connections. This is an enlarged view of Figure 6F.
